# Intramedullary Wire-Guided Bone Transport of the Ulna: A Case Report

**DOI:** 10.7759/cureus.98582

**Published:** 2025-12-06

**Authors:** Mohammad Fakhriy Badrol Hisham, Ahmad Arieff Atan, Norhaslinda Bahaudin, Abdul Muttalib Bin Abdul Wahid

**Affiliations:** 1 Department of Orthopaedics, Hospital Tuanku Ja'afar Seremban, Negeri Sembilan, MYS; 2 Department of Orthopaedics, Advanced Musculoskeletal Trauma and Limb Reconstruction Surgery Unit, Hospital Tuanku Ja'afar Seremban, Negeri Sembilan, MYS

**Keywords:** bone transport, forearm bone defect, intramedullary wire-guided bone transport, lrs external fixator, open fracture ulna

## Abstract

Diaphyseal bone defects of the forearm may arise from high-energy trauma, the surgical excision of devitalised or contaminated bone in open fractures, tumour resection, or extensive debridement for infected non-unions. These defects pose a considerable challenge in trauma and orthopaedic surgery due to the complexity of restoring limb function and structural integrity. Distraction osteogenesis, particularly via the Ilizarov technique, has become a preferred method among orthopaedic surgeons, owing to its advantages, including preservation of soft tissue, reduced infection risk, and the ability to maintain joint mobility during treatment. This case report demonstrates the successful application of the bone transport technique via distraction osteogenesis using a mini limb reconstruction system (LRS) external fixator in the management of an open diaphyseal forearm fracture with bone loss, resulting in favourable radiological bone healing and excellent functional recovery.

## Introduction

Open diaphyseal forearm fractures with bone loss commonly result from high-energy trauma. Proper management of these open fractures is essential to restore the biomechanical function of the wrist and elbow, ensuring the preservation of supination, pronation, and overall joint motion [1]. Reconstructing these types of bone defects necessitates a stable fixation and an environment free of infection. The numerous available reconstruction techniques highlight the challenges involved in promoting healing across the bone loss area. Commonly used approaches include distraction osteogenesis via segmental bone transport, bone grafting with either autografts or allografts, and both vascularized and non-vascularized bone transfers [2]. We describe an interesting case of a patient who sustained an open fracture of the right radius and ulna, complicated by substantial segmental bone loss of the ulna. Given the severity of the injury, we opted for bone transport via distraction osteogenesis. This technique was chosen because it offers a reliable way to restore bone continuity without requiring bone grafting, which can introduce additional complications such as infection or donor site morbidity. The segmental defect was addressed using intramedullary wire-guided bone transport, facilitated by a mini limb reconstruction system (LRS) external fixator. This device was chosen because it provides enhanced soft tissue protection and allows for a more controlled and efficient bone transport process compared to circular fixators.

## Case presentation

A 14-year-old male patient was involved in a motor vehicle accident and presented to the emergency department with active bleeding and visible deformity over the distal third of the right forearm. On initial assessment, the patient was haemodynamically stable. A thorough physical examination revealed a puncture wound located over the distal third of the right forearm on the ulnar aspect, accompanied by a visible limb deformity. Despite the external injury, neurovascular assessment confirmed that the right upper limb remained intact. An immediate closed manipulative reduction was performed in the emergency department to address the deformity, and the limb was immobilised using a full-length backslab.

**Figure 1:**
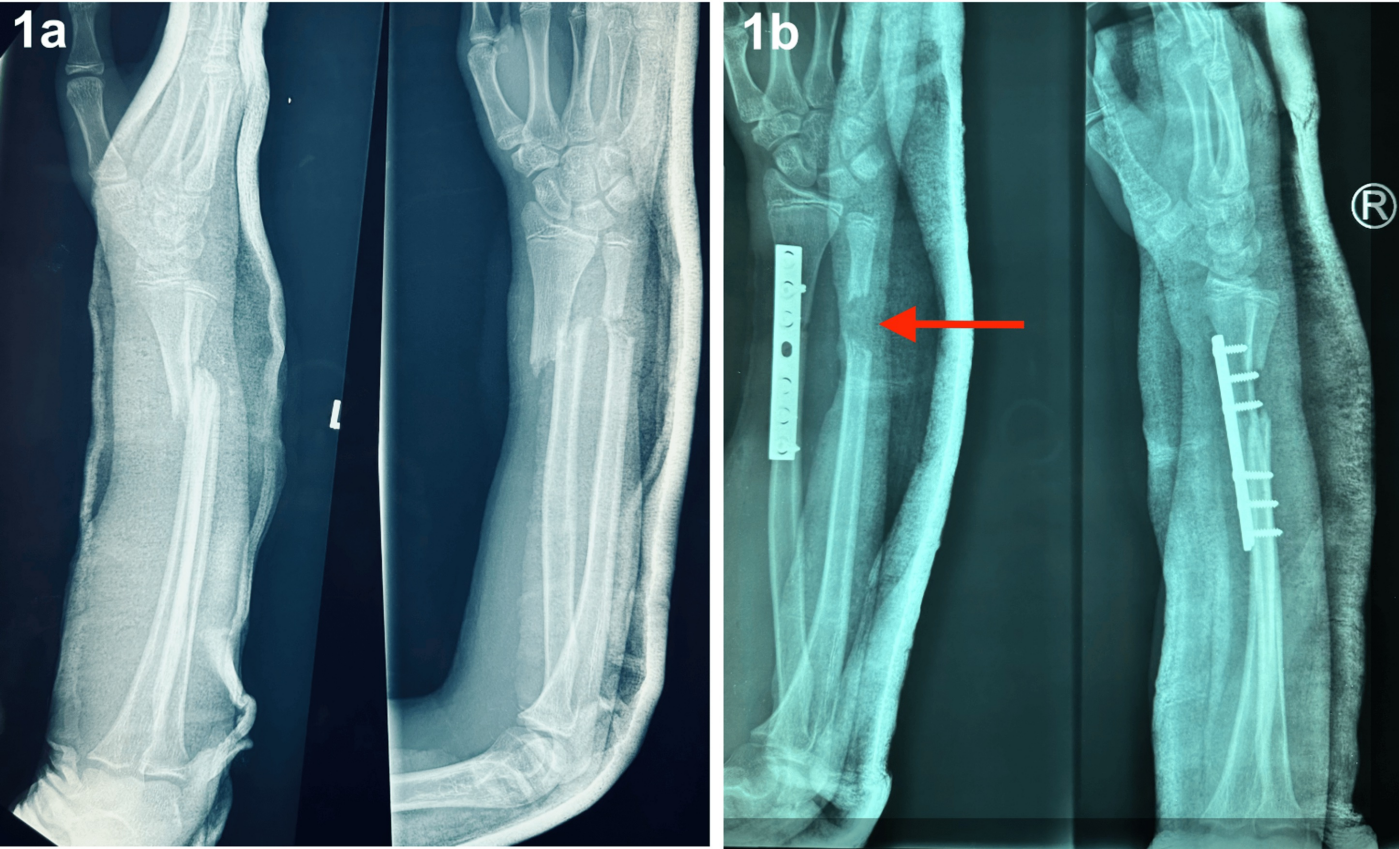
Plain radiographs in anteroposterior and lateral views showing a fracture of the distal third of the right radius and ulna with volar displacement (1a), and postoperative radiographs confirmed the presence of bone loss at the ulnar site (1b). Throughout the treatment course, potential complications such as pin-site infections were monitored, with no significant infections or pain reported.

Based on plain radiographs of the right forearm demonstrated fractures involving the distal third of both the radius and ulna, without any apparent disruption of the distal radioulnar joint (Figure 1a), a diagnosis of an open fracture of the distal third of the right radius and ulna was made. Intravenous cefuroxime was initiated promptly as part of the standard open fracture protocol. The patient was taken to the operating theatre within six hours of the injury for wound exploration, surgical debridement, and internal fixation. Intraoperatively, the injury was classified as a Gustilo-Anderson type IIIA open fracture. The radius was successfully stabilised using a small dynamic compression plate. However, fixation of the ulna was not feasible due to a segmental bone loss of approximately 2 cm identified intraoperatively. Following the procedure, the limb was immobilised with a backslab, and a plan was made for subsequent bone transport of the ulna using a mini LRS external fixator. Postoperative radiographs confirmed the presence of a bone loss at the ulnar site (Figure 1b).

**Figure 2:**
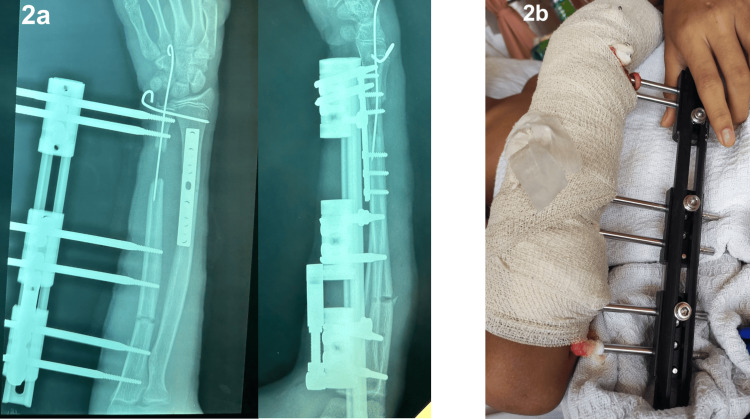
Postoperative radiograph in anteroposterior and lateral views of the right forearm (2a) and postoperative clinical image of the right forearm after the application of the mini limb reconstruction system external fixator for bone transport (2b).

Approximately 12 days following the initial procedure, bone transport of the right ulna was performed using a mini LRS external fixator, once the condition of the soft tissue and skin was deemed suitable for further intervention. Intraoperatively, six hydroxyapatite-coated pins were inserted into the ulnar segments to provide a stable monolateral external fixation. A corticotomy was carried out at the proximal third of the right ulna to initiate the bone transport process. Additionally, an intramedullary guide wire was introduced from the distal end of the ulna, extending into the middle bone segment, to assist in maintaining proper alignment throughout the bone transport process. While this technique is effective, potential risks include the breakage of the fine wire within the canal, which could compromise the alignment and progression of bone transport. Regular monitoring was essential to mitigate this risk, and no such incidents occurred in this case (Figure 2).

**Figure 3:**
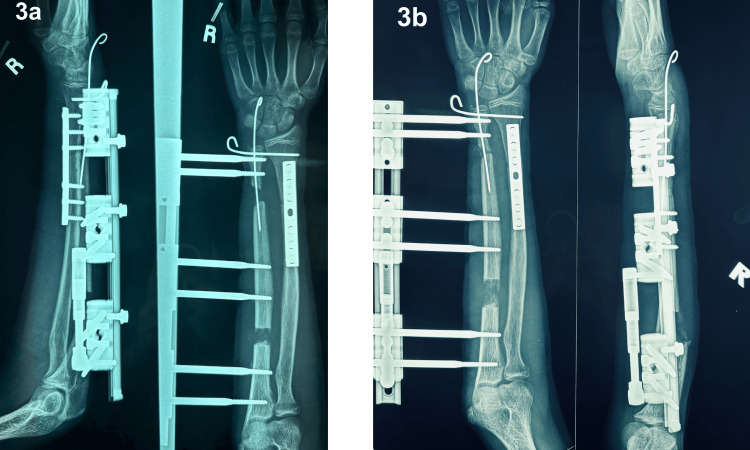
Radiographs of the right radius and ulna during the distraction phase, on day 10 (3a) and day 20 (3b), showing gradual bone transport and healing progress.

The distraction phase was initiated following a 10-day latency phase, with a gradual progression at a rate of 1 mm per day. This rate was chosen to optimise bone regeneration while minimising the risk of joint stiffness or delayed union (Figure 3). Throughout this phase, the patient underwent continuous, intensive upper limb physiotherapy aimed at preserving joint mobility and maintaining optimal hand function.

**Figure 4:**
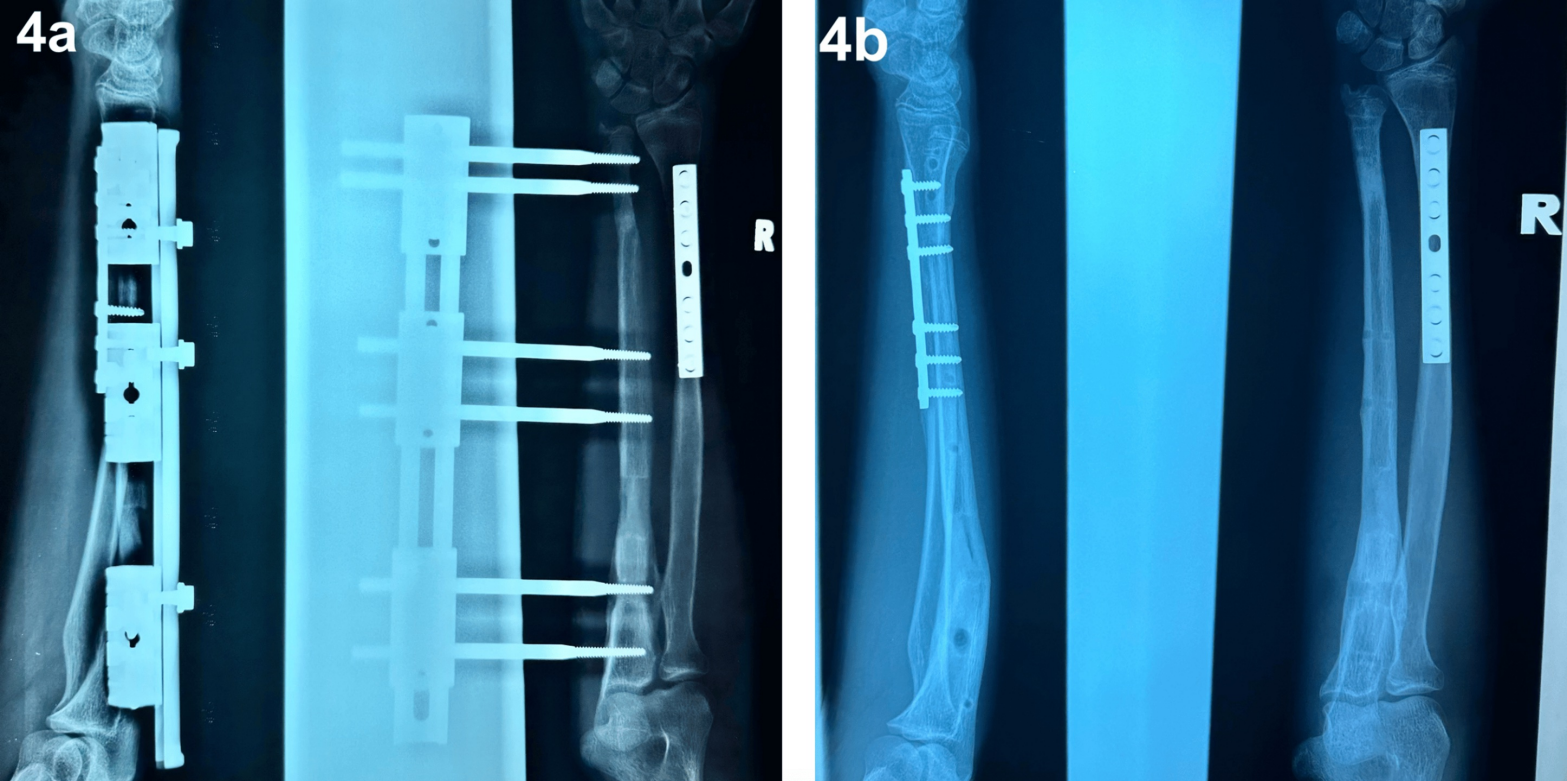
Radiograph of right radius and ulna in anteroposterior and lateral views on the day of mini limb reconstruction system removal (4a) and at one month following limb reconstruction system removal (4b).

At approximately four months postoperatively, the mini LRS external fixator was removed following the successful completion of both the distraction and consolidation phases. Serial radiographs demonstrated satisfactory bone alignment and adequate consolidation at the defect site, confirming that the objectives of the bone regeneration process had been achieved (Figure 4). Throughout the treatment course, there were no complications such as pin site loosening, infections, or pain reported.

**Figure 5:**
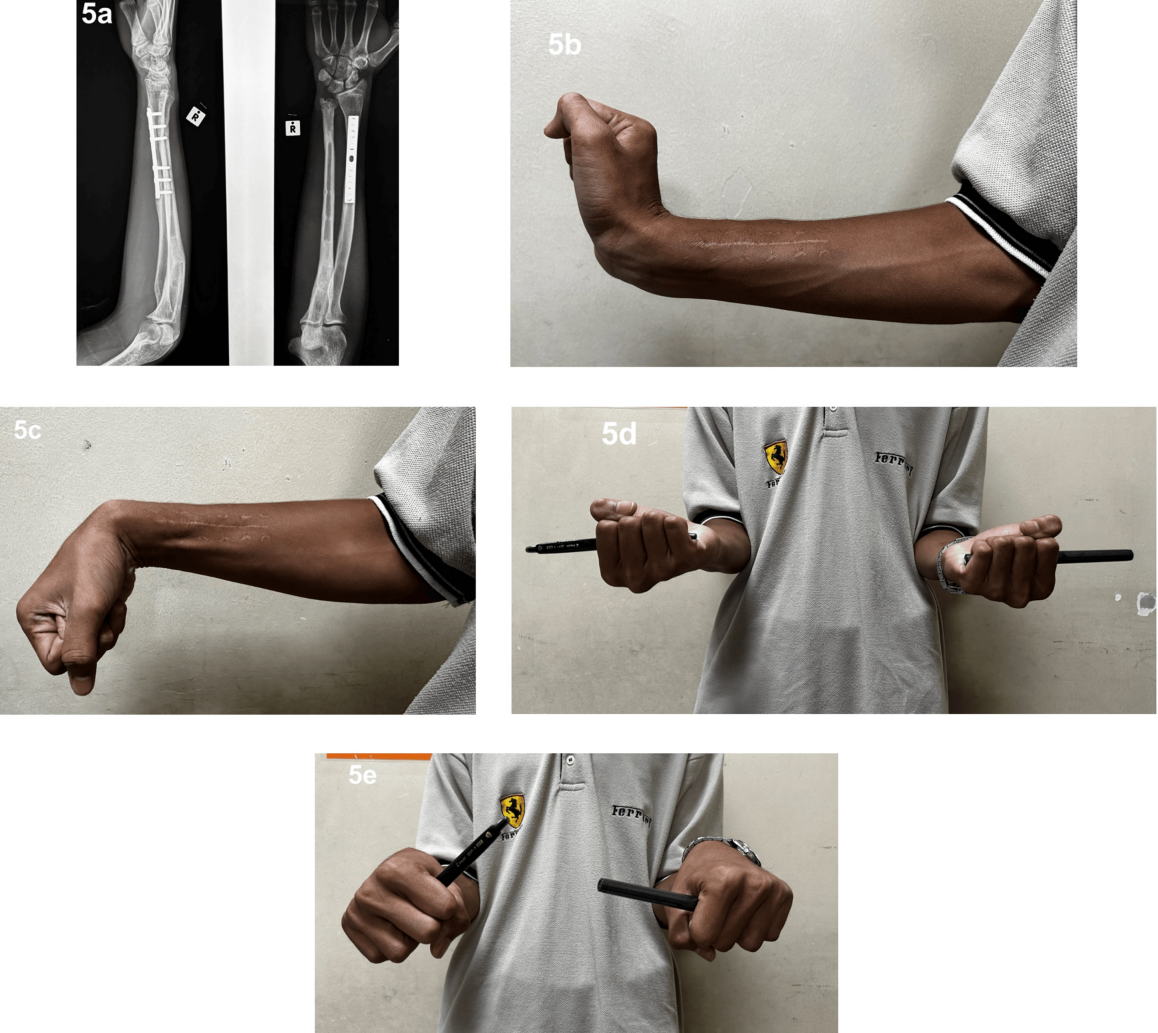
Postoperative radiograph in anteroposterior and lateral views demonstrating solid bony union with satisfactory anatomical alignment (5a); clinical image showing wrist extension up to 80 degrees (5b); wrist flexion up to 70 degrees (5c); full supination up to 90 degrees (5d); and pronation up to 45 degrees (5e).

At the one-year postoperative follow-up, radiographic imaging demonstrated a solid bony union at the site of the previous defect, with full consolidation and alignment achieved (Figure 5a). The patient's functional recovery paralleled the radiological findings, supporting the effectiveness of the bone transport method (Figures 5b-5e).

## Discussion

The forearm functions as a biomechanically integrated unit rather than as two separate bones, with coordinated movement relying heavily on the integrity of both bone and soft tissue structures [1]. This anatomical interplay is particularly critical for the execution of pronation and supination, which are essential for upper limb function [1]. In the present case, the identification of a segmental diaphyseal defect intraoperatively posed a considerable reconstructive challenge. Such defects disrupt the native biomechanics of the forearm and complicate efforts to restore the structural stability and functional range of motion. Effective definitive management requires careful consideration of both the mechanical and biological aspects of reconstruction to optimise the outcomes.

Reconstructive techniques for segmental bone defects are well-established in the context of lower limb injuries; however, literature on similar defects in the forearm remains limited. Bone transport, specifically through distraction osteogenesis, was chosen in this case due to its ability to restore bone continuity without the need for bone grafts, which can be scarce, cause donor site morbidity, and introduce infection risks. Various techniques in managing diaphyseal forearm bone defects that have been reported include interposition bone grafting, the induced-membrane (Masquelet) technique, fibular bone grafting, segmental bone transport, and a single-bone forearm creation via cross-union [3]. Bone transport via distraction osteogenesis offers a reliable and unique technique in managing segmental bone defects caused by trauma, infection, or congenital conditions. Compared to other techniques like autografting or the Masquelet technique, bone transport offers the advantage of not requiring an additional source of graft material, thus reducing the risk of complications associated with graft harvesting and donor site morbidity. This method involves performing an osteotomy, followed by the gradual mechanical separation of the bone ends using an external fixator, allowing regeneration of new bone within the created gap [4]. One of its main advantages is the ability to restore the bone continuity without the need for bone grafts, making it favourable when the source of grafts is limited [4]. Despite the effectiveness of distraction osteogenesis, managing potential complications is crucial. This procedure requires continuous monitoring to address issues such as infection, delayed union, and joint stiffness, particularly with the use of external fixators. Regular follow-up and adjustment of the fixator were essential in ensuring the success of the treatment, particularly in the context of maintaining optimal bone transport and joint motion [4].

In contrast, the Masquelet technique has proven to be a reliable and practical approach in managing large segmental bone defects, particularly those arising from trauma or infection. One advantage of this technique is that it does not require complex or specialised equipment, making it suitable for use in various clinical settings [5]. This technique helps to preserve the space needed for bone regeneration, reduces the risk of graft resorption, and prevents soft tissue from invading the dead space [5]. Additionally, it provides a shorter treatment duration compared to other reconstructive options, which may help minimise complications such as premature bone union or pin-site infections that are often seen with external fixation [5]. This method is typically performed in two stages, with the first stage involving a thorough debridement and insertion of a bone cement spacer, which promotes the formation of a biologically active membrane [5]. In the second stage, this cement spacer is removed and replaced with a bone graft tailored to the size of the defect [5]. However, a known limitation of this technique is the limited amount of autologous bone that can be harvested from donor sites such as the iliac crest, as harvesting large volumes may increase the risk of donor site complications, including pain and infection [5]. Therefore, the selection of an appropriate reconstructive approach must be tailored to the individual patient, taking into account factors such as defect size and location, soft tissue condition, patient age, and functional requirements.

In this case, the options of management were discussed between the attending surgeons, the patient, and the parents. We opted for bone transport using a mini LRS external fixator with intramedullary guided wire after considering several clinical factors, including compromised soft tissue condition from the initial trauma, which could have delayed definitive surgery if internal fixation was selected. Furthermore, internal fixation combined with bone grafting in the same operative setting posed a higher risk of infection (about 7% rate of infection in the Gustilo-Anderson type III group [6]), given the patient’s history of debridement and compromised soft tissue following the initial open fracture. Alternatively, if internal fixation with delayed bone grafting (Masquelet technique) had been chosen, the patient would have required two separate surgical stages and a waiting period between procedures, which could have potentially disrupted their school schedule. The need for autologous bone grafts also carries the risk of donor site complications, including pain and infection, which further influenced our decision to proceed with bone transport in this case.

Bone transport can be achieved by using various types of external fixation; however, the choice of device plays a critical role in preserving the forearm function. Although the circular external fixators, such as the Ilizarov system, are widely used in managing upper limb length discrepancies, their application in the forearm is associated with a higher risk of neurovascular injury, disruption of muscle-tendon movement, and restriction of pronation and supination of the forearm [7]. In this case, a monolateral LRS external fixator was preferred over the circular Ilizarov external fixator system for bone transport due to its several advantages. The LRS provides superior soft tissue management by minimising further trauma to the zone of injury, thus preserving the integrity of the soft tissues and surrounding neurovascular structures, while simultaneously maintaining the forearm motions [8]. This system also allows for a single-stage definitive surgical approach, combining the application of the LRS, guided wire, and corticotomy in one setting, which simplifies the overall treatment [8]. Furthermore, the mini LRS external fixator has shown great promise in paediatric cases, as demonstrated by its effectiveness in this case. It enables controlled bone transport while minimising soft tissue disruption. The system's design, which reduces the risk of neurovascular injury and provides better soft tissue management, is particularly advantageous in paediatric patients, where minimising long-term complications is a priority [8]. The use of an intramedullary guide wire in this case provided a controlled trajectory for bone transport during the distraction phase, helping to maintain proper alignment and minimise the risk of angulation and rotational deformities. Considering these advantages, the mini LRS external fixator with intramedullary wire-guided bone transport proved to be an appropriate and effective option in managing this case.

At the one-year follow-up, the patient demonstrated a satisfactory range of motion of the forearm with the radiological evidence of solid bony union, indicating that this treatment approach was at least nearly perfect in achieving both anatomical alignment and functional restoration. Although this bone transport technique has demonstrated favourable outcomes in managing segmental bone defects of the upper extremity, factors such as the severity of the initial injury, the presence of infection, and the length of the bone defect can significantly influence the healing process [4]. In the present case, these considerations were carefully evaluated when selecting the treatment approach. It is important to note that, although bone transport has been well-documented in the lower limbs, its application in the forearm is not as extensively studied. Current literature is limited, with most evidence coming from small case series or isolated reports. Larger, multicentre studies would be valuable in providing a more comprehensive understanding of the long-term outcomes, complications, and success rates of this technique for forearm bone defects [4]. Further research and functional outcome studies are still needed to enhance the bone transport devices, particularly those designed to better address the specific needs of patients with post-traumatic upper limb bone defects. 

## Conclusions

In conclusion, the management of open diaphyseal forearm fractures with significant bone defects can be effectively achieved using the bone transport technique with a mini LRS external fixator. This method provides a reliable, less invasive alternative for complex forearm reconstructions, allowing for bone regeneration without the need for grafts. With successful functional recovery and minimal complications observed, this approach demonstrates promising outcomes, particularly in paediatric patients, and may offer a more versatile solution compared to traditional methods. Further studies are needed to solidify its application in diverse clinical settings.
